# New insights into domestication of carrot from root transcriptome analyses

**DOI:** 10.1186/1471-2164-15-895

**Published:** 2014-10-14

**Authors:** Jun Rong, Youri Lammers, Jared L Strasburg, Natasha S Schidlo, Yavuz Ariyurek, Tom J de Jong, Peter GL Klinkhamer, Marinus JM Smulders, Klaas Vrieling

**Affiliations:** Plant Ecology and Phytochemistry, Institute of Biology Leiden, Leiden University, PO Box 9505, Leiden, 2300 RA The Netherlands; Center for Watershed Ecology, Institute of Life Science and Key Laboratory of Poyang Lake Environment and Resource Utilization, Ministry of Education, Nanchang University, Nanchang, 330031 China; Department of Biology, University of Minnesota-Duluth, Duluth, USA; Leiden Genome Technology Center, Human and Clinical Genetics, Leiden University Medical Center, Postzone S4-P, PO Box 9600, Leiden, 2300 RC The Netherlands; Plant Research International, Wageningen UR, PO Box 16, Wageningen, 6700 AA The Netherlands

**Keywords:** Crop and wild relative, *Daucus carota*, Domestication gene, Gene expression difference, High-throughput sequencing, Single nucleotide polymorphism, Root transcriptome

## Abstract

**Background:**

Understanding the molecular basis of domestication can provide insights into the processes of rapid evolution and crop improvement. Here we demonstrated the processes of carrot domestication and identified genes under selection based on transcriptome analyses.

**Results:**

The root transcriptomes of widely differing cultivated and wild carrots were sequenced. A method accounting for sequencing errors was introduced to optimize SNP (single nucleotide polymorphism) discovery. 11,369 SNPs were identified. Of these, 622 (out of 1000 tested SNPs) were validated and used to genotype a large set of cultivated carrot, wild carrot and other wild *Daucus carota* subspecies, primarily of European origin. Phylogenetic analysis indicated that eastern carrot may originate from Western Asia and western carrot may be selected from eastern carrot. Different wild *D. carota* subspecies may have contributed to the domestication of cultivated carrot. Genetic diversity was significantly reduced in western cultivars, probably through bottlenecks and selection. However, a high proportion of genetic diversity (more than 85% of the genetic diversity in wild populations) is currently retained in western cultivars. Model simulation indicated high and asymmetric gene flow from wild to cultivated carrots, spontaneously and/or by introgression breeding. Nevertheless, high genetic differentiation exists between cultivated and wild carrots (*Fst* = 0.295) showing the strong effects of selection. Expression patterns differed radically for some genes between cultivated and wild carrot roots which may be related to changes in root traits. The up-regulation of water-channel-protein gene expression in cultivars might be involved in changing water content and transport in roots. The activated expression of carotenoid-binding-protein genes in cultivars could be related to the high carotenoid accumulation in roots. The silencing of allergen-protein-like genes in cultivated carrot roots suggested strong human selection to reduce allergy. These results suggest that regulatory changes of gene expressions may have played a predominant role in domestication.

**Conclusions:**

Western carrots may originate from eastern carrots. The reduction in genetic diversity in western cultivars due to domestication bottleneck/selection may have been offset by introgression from wild carrot. Differential gene expression patterns between cultivated and wild carrot roots may be a signature of strong selection for favorable cultivation traits.

**Electronic supplementary material:**

The online version of this article (doi:10.1186/1471-2164-15-895) contains supplementary material, which is available to authorized users.

## Background

Understanding the molecular basis of crop domestication, especially identifying target genes under selection during domestication, can provide insight into the processes of rapid evolution and crop improvement [1–3]. The transcriptome represents all mRNA transcripts of actively expressed genes. Identifying sequence variants (e.g. single nucleotide polymorphisms: SNPs) and detecting differential gene expression patterns in transcriptomes is of primary interest in any attempt to characterize the effects of selection and identify target genes under selection [4]. The rapid development of high-throughput sequencing technology enables us to perform genome/transcriptome-scale studies not only by re-sequencing a few model species but also by *de novo* sequencing of many non-model species. This makes it feasible to compare the genome/transcriptome of a wide range of crops and progenitor species, permitting more solid conclusions to be drawn about the effects of domestication and revealing domestication genes. In this study, carrot was used as a model species to demonstrate how to study the effects of domestication and identify domestication genes based on transcriptome analyses.

Cultivated carrot (*Daucus carota* L. ssp. *sativus*) is one of the most popular vegetables in the world, providing the main source of dietary provitamin A [5–7]. According to the pigmentation of the roots, cultivated carrot can be distinguished into two main groups: the anthocyanin or eastern-type carrot (e.g. yellow or purple carrot), and the carotene or western-type carrot (e.g. yellow, orange or red carrot) [5]. For human consumption the eastern-type carrot has nowadays been largely replaced by the western-type carrot [5]. It is generally agreed that the eastern-type cultivated carrot originated in southwestern Asia in the area around Afghanistan only about 1100 years ago [5, 7]. However, the origin of the western-type cultivated carrot is still uncertain. Banga [8] demonstrated that an orange-colored carrot similar to the “Long Orange”-type western carrot first appeared on Dutch paintings in the beginning of the 17th century, suggesting a Dutch origin of the western orange carrot, probably directly selected from yellow eastern carrots. The Netherlands was the center of carrot breeding during the 18th century, and most of the modern varieties of western cultivated carrot may descend from the old orange Dutch carrots [7–9]. Because of the huge differences in root and leaf traits between eastern and western carrots, Heywood [5] disagreed with the idea that western carrot originated directly from eastern carrot. By summarizing the morphological evidence from different studies, he proposed a secondary domestication event, namely that the western cultivated carrot was selected from hybrids among yellow eastern carrots, cultivated white-rooted derivatives of wild carrot (*D. carota* L. ssp. *carota*) and adjacent wild populations of *D. carota* subspecies [5]. Iorizzo et al. [10] reported the first molecular study on carrot domestication indicating that eastern cultivated carrots originated in Central Asia and western cultivated carrots may have directly originated from eastern carrots. They focused mainly on wild carrot *D. carota* ssp. *carota*. However, other wild *D. carota* subspecies may also have played important roles in carrot domestication, because different *D. carota* subspecies within the *D. carota* complex can successfully hybridize in nature and the taxonomy is much disputed [5]. Therefore, in this study, various *D. carota* subspecies from different geographic regions will be used to further investigate the process of carrot domestication.

Usually domestication decreases the genetic diversity of crops through genetic bottlenecks and selection [1]. For instance, maize has only about 57% of the genetic diversity found in its progenitor [11]. In contrast, two previous studies found that carrot domestication did not result in a significant reduction of genetic diversity using allozymes, amplified fragment length polymorphisms (AFLPs) and inter-simple sequence repeat (ISSR) markers [12, 13]. However, the conclusions of these studies were based on only small regions of the carrot genome. Using thousands of SNPs, a new study by Iorizzo et al. [10] also detected similar levels of genetic diversity between cultivated and wild carrots suggesting the absence of a genetic bottleneck during carrot domestication. Considering the predominantly outcrossing nature of carrots and the relatively short time period of carrot domestication, the effects of domestication bottlenecks on cultivated carrots may have been offset by a high level of introgression from wild carrot and other *D. carota* subspecies after the bottlenecks. Further studies are required to test the hypothesis using different domestication models.

Key genes underlying valuable cultivation traits are mostly unknown in carrots. Since not all genes are targeted in domestication and/or breeding processes, we need to focus on those influencing favored traits to identify key genes under selection [1]. In the case of carrot, as a root crop, most of the traits of interest are related to the root, such as root color, shape, size, flavor etc. [5, 7]. Cultivated carrot differs from wild carrot in forming relatively large, unbranched, smooth and juicy storage roots with high sugar and carotenoid contents [5–7, 14]. The main varietal groups of cultivated carrot in use today are categorized by root type according to root shape, size and color [7]. Examples include the European carrot groups “Amsterdam Forcing”, “Berlicum”, “Chantenay”, “Flakkee”, “Nantes” and “Paris Market” [7]. Thus, the variation in the root transcriptomes between cultivated and wild carrots may provide essential information about the differentiation of cultivated carrot from wild carrot.

Against this background, the objectives of our study were:

1. To develop SNP markers polymorphic in the transcriptomes within and between diverse cultivated and wild carrots;
2. To infer the origin of cultivated carrot based on validated SNPs;
3. To show the effects of domestication on genetic diversity in the transcriptome;
4. To reveal gene expression changes between cultivated and wild carrots and identify key functional genes under selection.

As most of the domesticated traits may be related to the expression of functional genes in carrot roots, we sequenced and compared the root transcriptomes of several cultivated and wild carrots. SNPs were discovered and validated using diverse cultivated carrots, wild carrots and other wild *D. carota* subspecies. Phylogenetic analysis was performed to infer the origin of the cultivated carrot with different *Daucus* species as outgroup. Genetic diversity was calculated to evaluate the effects of domestication on genetic diversity. Domestication models were constructed to simulate the processes of carrot domestication. Key functional genes underlying cultivation traits were identified based on differential gene expression patterns between cultivated and wild carrots.

## Methods

### Plant materials

**Figure 1:**
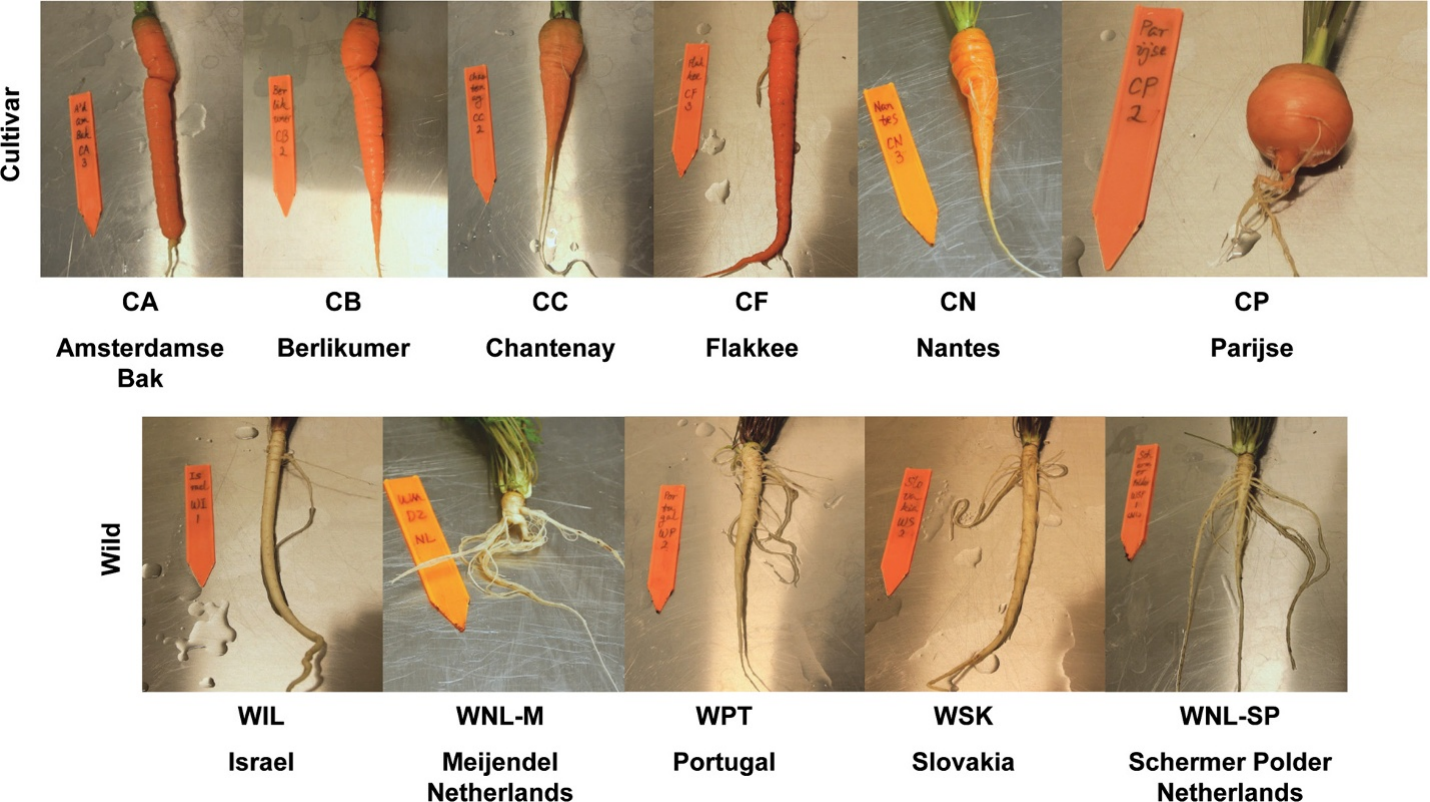
**Cultivated and wild carrot roots used for the transcriptome sequencing in the study.**

In order to discover representative SNPs with low ascertainment bias that could be used to represent the patterns of genetic diversity of cultivated and wild carrots, six varieties of cultivated carrot representing all European carrot root types and five wild carrot populations from widely dispersed sites were used (Figure 1 and Table 1). Seeds were germinated in Petri dishes on filter papers moisturized with water at room temperature for 2 weeks. To include more genetic diversity, three seedlings were randomly chosen from each cultivated carrot variety or wild carrot population (except for WPT, of which two seedlings were included). Each seedling was planted into a 15 × 15 × 20 cm^3^ pot with 1:1 mixed sand and soil. All plants were grown in a climate chamber with 16-h day/8-h night, temperature 20°C and relative humidity 70% for 11 weeks. Each root was carefully harvested to limit damage, quickly cleaned with water, transversely cut in the middle of the main root into small slices and immediately put into RNase-free tubes (about 100 mg per tube). All samples were freshly frozen in liquid nitrogen and stored at -80°C.

**Table 1:** **Number of reads and mean coverage to the reference sequence of cultivated and wild carrot transcriptomes**

Lane	ID	Sample name	Number of reads	Mean coverage
Cultivated carrots	1	CA (Amsterdamse Bak)^1^	3,774,122	14.4
(*D. carota* ssp. *sativus*)	2	CB (Berlikumer)	2,471,568	9.0
	3	CC (Chantenay)	10,969,116	36.2
	4	CF (Flakkee)	11,973,958	42.5
	5	CN (Nantes)	10,462,118	34.3
	6	CP (Parijse)	15,686,674	51.8
Wild carrots	7	WIL (Lachish, Israel: 31.565°N, 34.849°E)^2^	1,353,622	4.6
(*D. carota* ssp. *carota*)	8	WNL-M (Meijendel, Netherlands: 52.156°N, 4.380°E)	137,338	0.5
	9	WPT (Esposende, Portugal: 41.533°N, 8.783°W)	11,685,548	36.7
	10	WSK (Trenčin, Slovakia: 48.892°N, 18.037°E)	8,352,412	24.9
	11	WNL-SP (Schermer Polder, Netherlands: 52.621°N, 4.861°E)	16,706,796	51.3

^1^Variety names are given in parentheses.

^2^Locations of wild carrots are given in parentheses.

To further validate the SNPs and infer the origin of cultivated carrots, an additional set of 49 cultivated carrots with both eastern and western cultivars, 18 wild carrots (*D. carota* ssp. *carota*), 32 accessions of 10 other wild *D. carota* subspecies, and 6 accessions of 4 different wild *Daucus* species (*D. muricatus*, *D. aureus*, *D. guttatus* and *D. broteri*) from Mediterranean, Southern, Western and Northern Europe, Western, Central, Southern and Eastern Asia were used (Additional file 1: Table S1).

### RNA extraction and purification

RNA was extracted from each root sample with the RNeasy Plant Mini Kit (QIAGEN, Venlo, The Netherlands). About 2000 ng RNA was taken from each sample and adjusted to a volume of 12 μL with RNase-free water. For DNA digestion, this was mixed with RNase-free 1.5 μL 10× DNase I reaction buffer, 0.75 μL of 2 U/μL DNase I (Ambion) and 0.75 μL water to a total volume of 15 μL. The mixture was placed at room temperature for 15 min. To inactivate DNase I, 1.5 μL RNase-free 25 mM EDTA was added to the mixture, which was then incubated at 65°C for 10 min. Subsequently, the three RNA samples of plants of the same cultivated carrot variety or wild population (two samples for WPT) were equimolarly pooled and adjusted to a volume of 100 μL with RNase-free water. The RNA was purified with the RNeasy Mini Kit (QIAGEN, Venlo, The Netherlands). The RNA samples were stored at -80°C.

### Transcriptome sequencing (RNA-Seq)

RNA-Seq analysis was performed at Leiden Genome Technology Center (LGTC). First, cDNA fragments were synthesized and amplified from each RNA sample with the Ovation RNA-Seq System (NuGEN). Then, sample preparation for Illumina multiplexing paired-end (PE) sequencing was performed according to the Illumina protocol. Each sample was tagged with a unique index tag (Index primer 1–11 for sample ID 1–11 in Table 1), permitting discrimination of sequences from different samples after multiplex sequencing. The quality and quantity of each sample was measured with an Agilent 2100 Bioanalyzer (Agilent Technologies). Each sample was diluted to 10 nmol/L. We then equimolarly pooled cultivated carrot samples into one tube and wild carrot samples into another for sequencing. Cluster generation was performed with the pooled cultivated carrot sample in one lane of the Illumina flow cell and the pooled wild carrot sample in another. The PE sequencing was carried out on the Illumina Genome Analyzer IIx for 75 cycles.

### Sequence assembly and mapping

The default Illumina pipeline filter (chastity ≥0.6) was used for cleaning up raw reads. CLC Genomics Workbench 4.0 (CLC bio) was used for a *de novo* assembly (Insertion cost = 3; Deletion cost = 3; Mismatch cost = 2) of all obtained sequences from both cultivated and wild carrots into contigs. All resulting contigs with a coverage ≥40 or length ≥500 bases were selected and concatenated to create a single consensus reference sequence. The coverage of at least 40 was chosen in order to obtain coverage of at least 3–4 per transcript per sample. This allowed us to genotype each sample and compare gene expressions between samples later. In the reference sequence, adjacent contigs were separated by a 30-letter string of 10 Ns, 10 Cs, and 10 Ns. This artificial spacer sequence was designed not to disturb read alignment at the end of the contig. Then, reads from each cultivated or wild carrot were aligned to the reference sequence with the program Burrows-Wheeler Aligner (BWA) [15]. The alignments were processed in the Sequence Alignment/Map (SAM) format with the program SAMtools [16]. Afterwards the alignment data were processed in R (version 2.12.1) [17] for additional quality control, for genotyping each cultivated carrot or wild carrot population, for SNP discovery and for further statistical analysis.

### SNP calling

For SNP discovery, positions in the reference sequence were selected for those reads that were present in all samples. We did not include the reads of WNL-M in this screening because the number of reads was 10–100 fold less than that of the others (Table 1). Second, positions with more than 1 base ‘N’ in a sample were removed. If more than two different nucleotides were observed at a given position in a sample, only the most- and the second-most-observed nucleotides were considered as real alleles and the number of remaining nucleotides was used to calculate the error rate (*ϵ*) per nucleotide (A, T, C, or G):
1

where *n*_*1*_ is the number of the most-observed nucleotide, *n*_*2*_ is the number of the second-most-observed nucleotide and so on. The value of *ϵ* was generally very low: 75.7% positions with mean *ϵ* = 0 and 97.6% with mean *ϵ* <0.05. That suggests high quality of the sequencing data at the selected positions. To reduce false positive rates, if *ϵ* ≥0.05 the sample was assigned an ‘N’ at the position. Otherwise, a genotype was identified according to the allele state. First, the maximum number of errors (*n*_*E*_) per nucleotide (A, T, C, or G) of a sample at a position was estimated as:
2

where *qbinom* is an R function calculating the quantile (in our case *p* = 0.99) of a binomial distribution with given number of reads *n* = *n*_*1*_ + *n*_*2*_ + *n*_*3*_ + *n*_*4*_ and error rate *ϵ*. If the observed number of a nucleotide was larger than *n*_*E*_, the chance of the observation due to error is smaller than 0.01 and it was taken into consideration as a valid allele. To reduce false positive rates, if the value of *ϵ* of a sample at a position (e.g. *ϵ* = 0) was less than the mean *ϵ* over all samples and positions, the mean *ϵ* was used for the calculation. If no nucleotide had a count larger than *n*_*E*_ or more than two nucleotides had counts larger than *n*_*E*_, the sample was assigned an ‘N’ at the position.

On the other hand, all samples but one (WPT contains two individuals) are a mixture of three individuals. Therefore, the number of reads (*n*) of a sample at a position should be at least 6 or 4 for genotyping (carrot is diploid) and if *n* <6 or *n* <4 (for WPT) the sample was assigned an ‘N’ at the position as well. Suppose different individuals of a sample have similar patterns of expression for the same gene. Then a sample contains heterozygous individual when:
3

or
4

where (*n*_*2*_ - *n*_*E*_) is the corrected number of nucleotides, which should be higher than the minimum expected number of nucleotides given the minimum ratio of an allele in the mixture (1/6 or 1/4), *n* and 0.01 in Equations 3 and 4 means that the chance of a value equal to or less than the expected value is no more than 0.01. Otherwise, the sample was scored as homozygous for the most-observed nucleotide. With the same strategy as indicated above, the genotypes of different samples at different SNP positions were scored. Finally, we selected for further analysis genotypes of SNP positions with no more than 1 ‘N’ genotype, at least one different genotype other than ‘N’ and no more than 2 alleles over all cultivated and wild carrot samples.

### SNP validation

The KBioscience Competitive Allele-Specific PCR (KASP) genotyping system (LGC KBioscience, UK) was applied for SNP validation. Primers were designed for 1000 SNPs based on sequences with 50 bases on either side of a SNP. Besides the carrot samples used for sequencing (10 × 3 + 1 × 2 = 32 samples), an independent set of 37 cultivated carrots, 15 wild carrots and 32 accessions of 10 other wild *D. carota* subspecies (part of the accessions in Additional file 1: Table S1) was used for SNP validation (116 samples in total). As a result, 622 SNPs were confirmed to be polymorphic. Afterwards, another 21 samples (indicated in bold in Additional file 1: Table S1) involving eastern-type carrots (as comparison to western carrots) and different *Daucus* species (as outgroup) were genotyped at 89 SNP positions, a subset of the 622 SNPs. Thus, we had two sets of genotypic data: 1) the 622-SNP dataset containing the genotypic data at 622 SNP positions of 115 carrot samples (WNL-SP3 was deleted for having too many missing data; without outgroup); 2) the 89-SNP dataset involving the data at 89 SNP positions of 136 samples (with outgroup).

### Genetic structure

A combined dataset of both the 622-SNP and 89-SNP datasets were used for the phylogenetic analysis, i.e. 115 samples genotyped at 622 SNP positions and 21 samples genotyped at 89 SNP positions. MrModeltest version 2.3 [18] was used for selecting the best-fit model of nucleotide substitution. The GTR + G model is the best-fit with the smallest Akaike information criterion (AIC) value and the highest Akaike weight. Then, a Bayesian estimation of phylogeny was performed using MrBayes version 3.1.2 from the CIPRES Science Gateway (http://www.phylo.org/portal2/tools.action) [19–21]. Population structure of cultivated carrots, wild carrots and other wild *D. carota* subspecies (using the 622-SNP dataset) was inferred using Structure 2.3.4 [22]. An admixture ancestry model was used and allele frequencies were assumed to be independent among populations. Population number (*K*) was set from 1–8. Three replicate runs were carried out for each *K*. Each run had a burn-in length of 50,000 iterations and 100,000 iterations after burn-in. Using the 622-SNP dataset, the *Fst* between cultivated and wild carrots was calculated with the software package ∂a∂i (dadi version 1.6.3) [23]. The 95% confidence interval (95% CI) of the estimate was inferred by resampling SNP positions (1000 bootstrap samples).

### Genetic diversity

The genetic diversity estimates were calculated using the 622-SNP dataset. The proportion of polymorphic loci (*P*) was calculated for cultivated carrots, wild carrots, and wild carrots plus other wild *D. carota* subspecies separately. A polymorphic locus is defined as having more than 1 allele. The 95% CIs of the *P* estimate were calculated from 1000 bootstrap samples of SNP positions. Nucleotide diversity (*θ*_*π*_), Watterson’s estimator of theta (*θ*_*w*_) and Tajima’s D of cultivated carrots, wild carrots, and wild carrots plus other wild *D. carota* subspecies were calculated with the software package ∂a∂i (dadi version 1.6.3) [23]. The 95% CIs of the estimates were inferred by resampling SNP positions (1000 bootstrap samples).

### Domestication model

**Figure 2:**
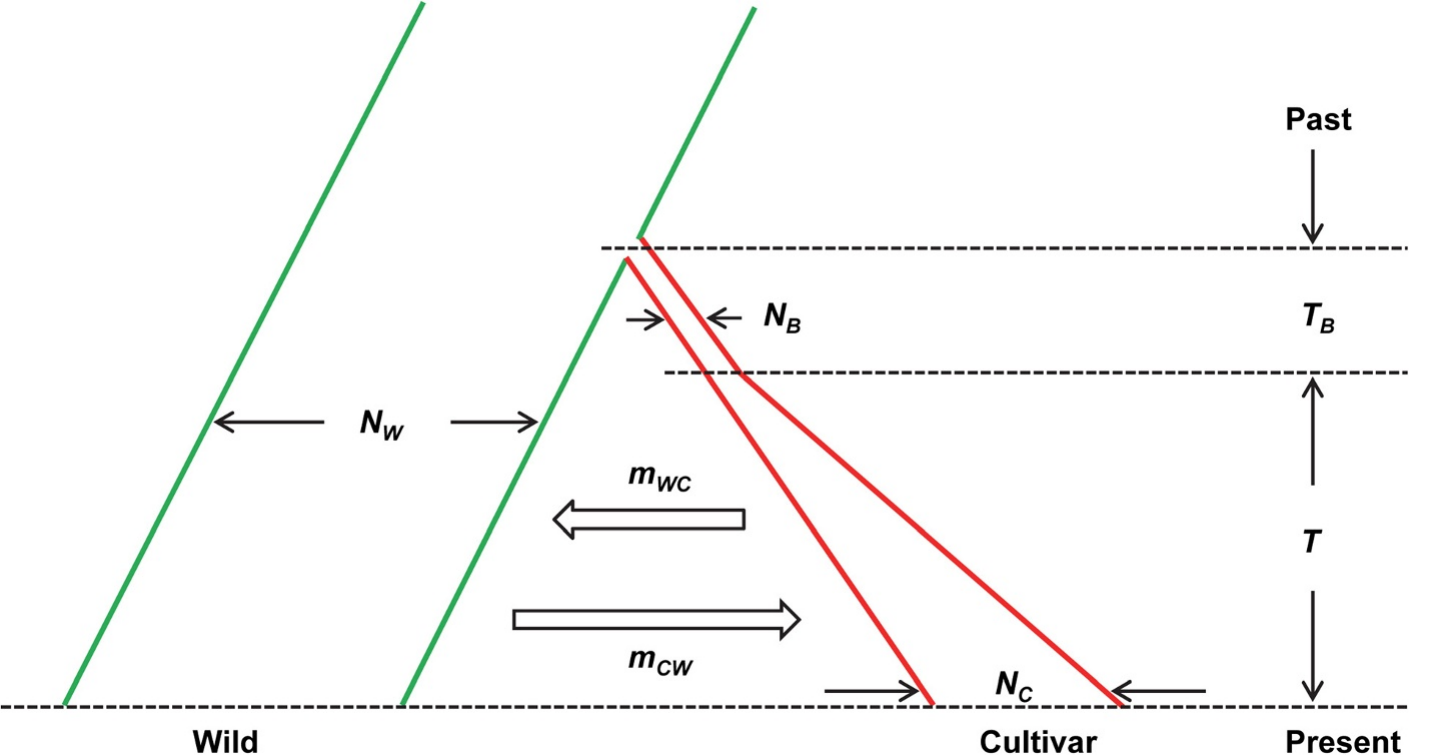
**Illustration of the domestication model.** The effective population size of wild carrot (*N*_*W*_) is constant. Carrot domestication started *T*_*B*_ + *T* generations ago. The size of domestication bottleneck is *N*_*B*_ and the duration of the bottleneck is *T*_*B*_. Afterwards, the effective population size of cultivated carrot increased exponentially. After *T* generations, cultivated carrot has a present population size of *N*_*C*_. During the past *T* generations, gene flow occurred between cultivated and wild carrots. The migration rate from cultivated to wild carrot is *m*_*WC*_ and that from wild to cultivated carrot is *m*_*CW*_.

The domestication model used is illustrated in Figure 2. When splitting from wild carrot about 1100 years ago, cultivated carrot was assumed to go through a bottleneck. Afterwards, the effective population size of cultivated carrot was assumed to increase exponentially, together with gene flow and introgression between cultivated and wild carrots (Figure 2). The model was used to fit SNP data of cultivated and wild carrots with the software package ∂a∂i (dadi version 1.6.3) [23]. The 622-SNP and 89-SNP datasets were used respectively. ∂a∂i is a powerful tool for fitting population genetic models to the joint allele frequency spectrum (FS) using a diffusion approximation [23]. It has been shown to be very efficient for estimating demographic parameters from genetic data and testing crop domestication models [24, 25]. Due to computational limitations, the two-dimensional FS of wild and cultivated carrots was projected down to the same smaller sample size of 10 by averaging over all possible re-samplings of the larger sample size data [23]. The 622-SNP dataset did not contain an outgroup to polarize SNPs, therefore we set polarized = False to ignore outgroup and fold the resulting FS. For the 89-SNP dataset, outgroup data were used to polarize the ingroup SNPs as ancestral or derived as long as there were at least four called outgroup SNPs, in which case the outgroup SNP at highest frequency was considered ancestral. Domestication models were constructed in Python scripts using the ∂a∂i package with parameters specified in Figure 2. Three models were tested: 1) no migration between cultivated and wild carrots (*m*_*WC*_ = *m*_*CW*_ = 0); 2) symmetric migration (*m*_*WC*_ = *m*_*CW*_ = *m*); and 3) asymmetric migration. The parameters were estimated by fitting models to the data and choosing the maximum likelihood values. The 95% CIs of parameter estimates were inferred by fitting data sets resampled over SNP positions.

### Putative genes under selection

Genes under selection may show very different expression patterns between cultivated and wild carrots. Because the total number of reads varied across samples (Table 1), we first normalized the coverage of contigs. Normalized gene expression was calculated as the coverage of a contig from a given sample divided by the mean coverage of all the contigs in the reference sequence from the sample (Table 1). Then, the difference in gene expression of a contig between cultivated and wild carrots was calculated as (mean coverage of cultivated carrots - mean coverage of wild carrots)/(mean coverage of cultivated and wild carrots). The 95% CIs of the mean gene expression difference were calculated from 1000 bootstrap samples of contigs. Genes represented by contigs with coverage from only cultivated or wild carrot were termed “unique expression”. Putative functions for these unique expression contigs were determined by BLAST (Basic Local Alignment Search Tool: http://blast.ncbi.nlm.nih.gov/) in Genbank.

## Results and discussion

For the high-throughput transcriptome sequencing, we obtained over 57 million reads from cultivated carrot roots, and over 40 million reads from wild carrot roots. 97% of the reads of cultivated carrot had tags and were assigned to one of the cultivated varieties, and 94% of the reads of wild carrot had tags and were assigned to one of the wild populations (Table 1). Each read was 75 bases long. 91% of the reads were assembled *de novo* into 252,715 contigs (mean length = 216; mean coverage = 122). 45,165 contigs were selected (coverage ≥40 or length ≥500; mean length = 411) representing the consensus/majority sequence of heterozygous and long contigs, and concatenated to form a single consensus reference sequence. The final reference sequence for the root transcriptome contained 18,600,079 bases (excluding artificial strings between contigs). The size of the protein-coding region in the carrot haploid genome (473 Mb) is estimated to be about 47.7 Mb [26]. The selected reference sequence of the root transcriptome therefore corresponds to the size of about 39% of the complete carrot transcriptome. 41% of the reads from cultivated carrots and 40% of those from wild carrots were aligned to the reference sequence. The mean coverage of the various cultivated carrots was 31.3 ± 6.7 (mean ± standard error), for the wild carrots this was 29.4 ± 9.9 (excluding WNL-M, with very low mean coverage). The selected reference sequence is therefore not expected to cause a significant bias in comparing the read alignments of cultivated and wild carrots. Further analyses were all based on the alignments to the selected reference sequence. 11,369 SNP positions were identified in the reference sequence. Considering the conservative method of SNP discovery (to reduce false positive rates), the true number of SNPs is most likely higher. The ratio of transition substitutions (32.2% A/G and 31.4% C/T) to transversions (11.4% A/C, 10.8% G/T, 7.8% A/T and 6.4% C/G) was about 1.75 to 1.

Primers were designed for testing 1000 SNPs in a KASP assay, of which 871 generated PCR products. Of these, 79 were monomorphic or had many unreliable data points in the sequencing samples. The unreliable data points may be due to mismatches of primers (e.g. flanking SNPs). 792 (79.2% of the total SNPs tested) showed the expected SNP patterns in the sequencing samples. In the independent set of cultivated carrots, wild carrots and other wild *D. carota* subspecies (Additional file 1: Table S1), 170 out of the 792 SNPs showed only one genotype for most samples or many unreliable data points, and 622 (62.2% of the total SNPs tested) were polymorphic. Iorizzo et al. published the first large-scale transcriptome of carrot in 2011 [27]. They computationally identified 20,058 SNPs [27]. However, only 60% of their 354 tested SNPs had the expected SNPs in their sequencing samples, and 14% of the 354 tested SNPs were polymorphic in an unrelated mapping population [27]. They sequenced the transcriptomes of three cultivated carrots and a pool of F4 RILs from a cross between cultivated and wild carrots [27], which may have led to ascertainment bias towards SNPs polymorphic in cultivated carrots. The higher success rate of our SNPs in both the sequencing and independent sets of samples indicates that the use of sequences from diverse cultivated and wild accessions together with a conservative SNP discovery method across these sequences have effectively reduced the false positive rate. Primers for the 622 validated SNPs are reported in Additional file 2: Table S2. They can be used for carrot genetic mapping and breeding as well as for population and evolutionary genetics studies.

### Genetic structure

**Figure 3:**
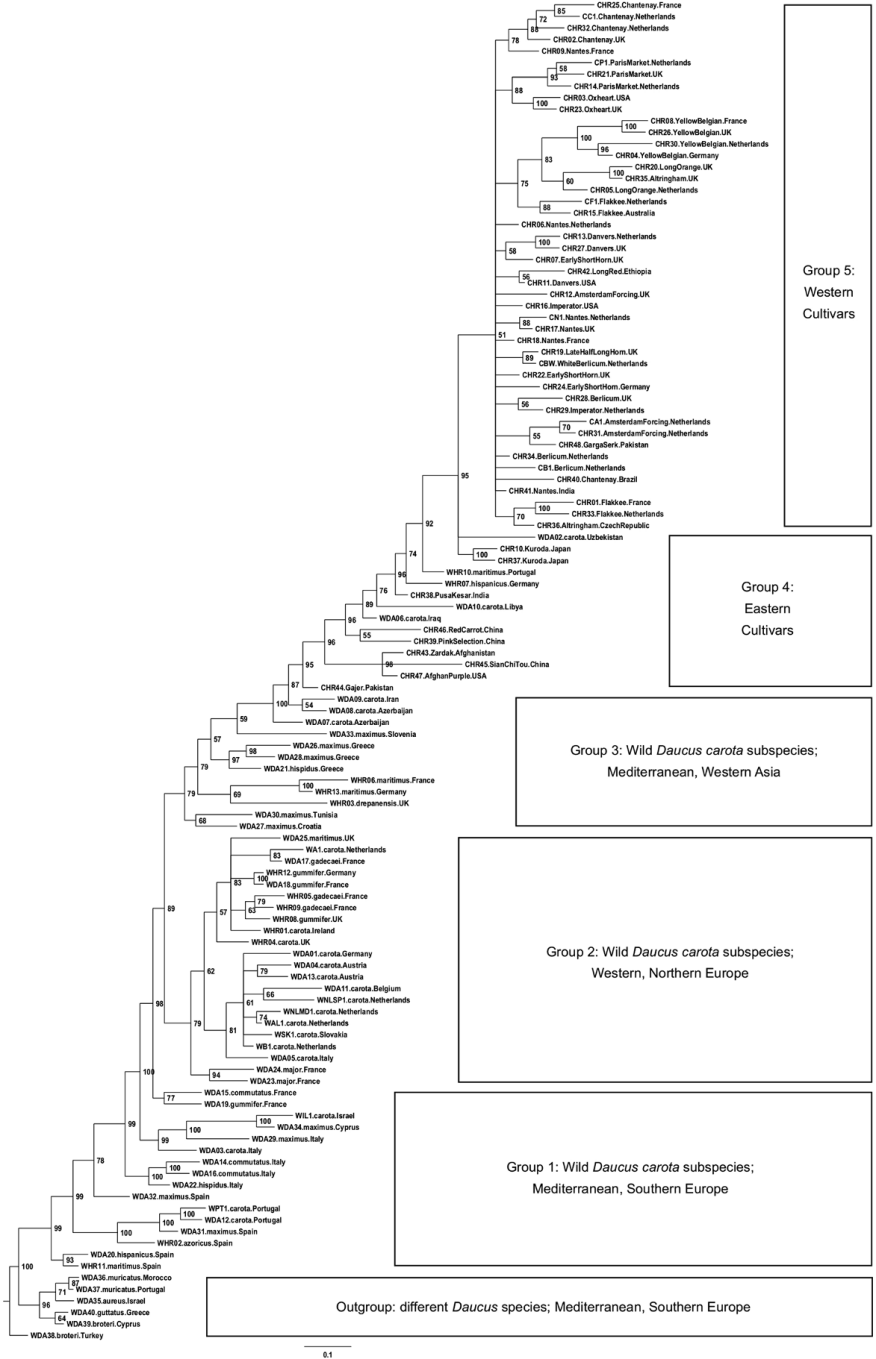
**Phylogenetic tree of carrot.** Phylogenetic analysis was based on the combined datasets of 622-SNP and 89-SNP. Different *Daucus* species were used as outgroup to *D. carota*. Numbers at the nodes indicate posterior probabilities (%). Sample names beginning with “W” are wild species and those with “C” are cultivars; the middle name of each sample indicates species name (for outgroup) or subspecies name of wild species, or root type/accession name of cultivars; the sampling country is indicated at the end. For more details of the samples see Table 1 and Additional file 1: Table S1. Group 1–5 were designed to represent the main phylogeographic structure of the tree. Note that the grouping is somewhat arbitrary because there is no distinct boundary between groups, for instance a few wild carrots are within the Group 4 of Eastern Cultivars.

**Figure 4:**
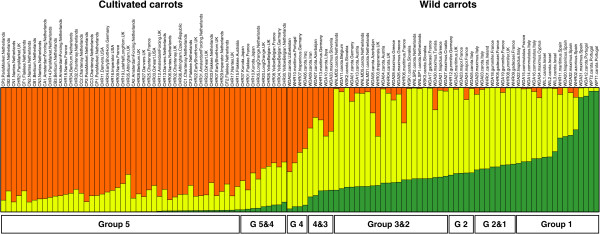
**Genetic structure of carrot.** Genetic structure of cultivated and wild carrots was inferred using Structure 2.3.4 based on the 622-SNP dataset. The clusters of *K* = 3 were shown for the highest Ln likelihood. Vertical bars represent different cultivated and wild carrots. The label of each sample is given above each bar. Those beginning with “C” are cultivars and with “W” are wild species; the middle name of each sample indicates root type/accession name of cultivars or subspecies name of wild *Daucus carota* subspecies; the sampling country is indicated at the end. For details of each carrot sample see Table 1 and Additional file 1: Table S1. The length of each colored segment in a bar represents the relative proportion of the Bayesian assignment to each cluster. Group 1–5 indicated below the bars are according to Figure 3.

Based on the genotypes at the validated SNP positions, a phylogenetic tree of carrot was constructed (Figure 3). The huge volume of data meant that a phylogenetic tree with a clear genetic structure could be drawn that could not readily be resolved using traditional methods [28]. Although the domestication of cultivated carrot is a relatively recent event [5, 7], and cultivated carrot can readily hybridize with wild carrot in nature owing to the high outcrossing potential [5, 14, 29, 30], most of the cultivated carrots are clearly separated from the wild carrots in our study demonstrating the strong effects of human selection. Western cultivars are nested within eastern cultivars, which are basal in the cultivated carrot clade. This pattern was also broadly supported by the clustering with the program Structure [22], where three populations (*K* = 3) had the highest Ln likelihood (Figure 4), and cultivated and wild carrots cluster in fairly distinct groups, although there is some evidence of introgression. The high *Fst* = 0.295 (95% CI: 0.282 - 0.309) between cultivated and wild carrots also indicates clear genetic differentiation between them. On the other hand, different wild *D. carota* subspecies are mixed together in the phylogenetic tree (Figure 3) as well as in the Structure clustering (Figure 4). *D. carota* ssp. *carota* did not form a distinct clade or cluster. These results are consistent with the previous findings that different subspecies within the *D. carota* complex can freely interbreed [5]. In addition, the results suggest that besides *D. carota* ssp. *carota* other wild *D. carota* subspecies may also have contributed to the domestication of cultivated carrots as was also pointed out in previous studies [5]. In the study of Iorizzo et al. [10], wild *D. carota* subspecies (other than *D. carota* ssp. *carota*) were clustered separately from wild carrots. However, the wild *D. carota* subspecies they used were from Portugal and France only [10]. The wild *D. carota* subspecies used in our study represent much more diverse geographic origins (9 European countries, 1 African and 1 Asian) (Additional file 1: Table S1) including a higher level of genetic diversity. This may explain the fact that wild carrots and other wild *D. carota* subspecies with similar geographic origins are clustered together in our study (Figure 3 and Figure 4). It is commonly recognized that the Mediterranean region may be the diversity center of *Daucus* species [5]. For *D. carota* subspecies, our study also showed that it most likely originated from the Mediterranean region and Southern Europe (Figure 3). From there, they spread to Western, Northern Europe and Western Asia (Figure 3).

The eastern-type cultivated carrots may have originated in the areas from Western to Central Asia (Figure 3), which is in close agreement to the results of Iorizzo et al. [10]. Their study indicated that cultivated carrots most likely originated in Central Asia [10]. With respect to the origin of the western-type cultivated carrots, our results strongly support that they were derived from eastern carrot cultivars, but introgression from wild carrots may have played a role as well, as proposed by Heywood [5]. The Structure clustering results imply that “Long Orange” may be the original root type of western-type orange carrots (CHR05 and CHR20 in Figure 4). Although the “Yellow Belgian” root type clusters closer to wild carrots, these accessions have white (CHR08 and CHR26) or yellow (CHR04 and CHR30) roots. The “Long Orange” type carrot was the first observed type of orange carrot on Dutch paintings as early as about 1600 [7, 8]. Thus, our results support the notion that the western-type orange carrot may have originated in The Netherlands prior to the 17th century. However, the phylogenetic analysis does not support this hypothesis (Figure 3). On the other hand, the Structure clustering in our study was based on cultivated and wild carrots primarily of European origin. While Turkey was regarded as one of the places of origin of western carrot in previous studies [5], our study did not include cultivated and wild carrots from Turkey. Therefore, a more detailed study involving more carrot samples from Middle East (e.g. Turkey) needs to be conducted to further determine the place of origin of western carrot.

### Effects of domestication on genetic diversity

For the validated 622 SNP positions, all genetic diversity estimates of cultivated carrot are significantly lower than those of wild carrot (Table 2). The genetic diversity estimates between wild carrot and wild carrot plus other *D. carota* subspecies are not significantly different (Table 2). Domestication has therefore significantly decreased genetic diversity in cultivated carrot, which may be due to genetic bottlenecks and/or selection, although the decrease is relatively small in absolute terms. Tajima’s D is significantly positive in both cultivated and wild carrots, although it is higher in cultivated carrot (Table 2), which could be due to genetic bottlenecks, population expansion after bottlenecks, balancing selection, and/or introgression. The insignificant reduction of genetic diversity found in previous studies of carrot domestication [12, 13] may be due to the low genetic variation in the allozyme markers and to the fact that only a small part of the carrot genome was under investigation, which may not have been under selection during domestication. However, our results are also somewhat different from those of a recent study by Iorizzo et al. [10], who found no difference in genetic diversity between cultivated and wild carrots using thousands of SNPs. The expected heterozygosity *H*_*e*_ of wild carrot (*D. carota* ssp. *carota*) within our 622-SNP dataset was higher than that estimated by Iorizzo et al. [10], which may be owing to the fact that the wild carrot accessions used in our study represent more diverse geographic origins (Additional file 1: Table S1). On the other hand, the *H*_*e*_ of cultivated carrot in our study was lower, which may be due to the fact that Iorizzo et al. [10] used more eastern cultivated carrots for genetic diversity estimate while we focused mainly on western orange carrot, primarily of European origin. Such a result suggests that the genetic diversity of western or European carrot may be lower than eastern carrot implying the origin of western carrot from eastern carrot. Another difference is that we used SNPs developed from genes that are expressed in the roots only, while Iorizzo et al. [10, 27] also included SNPs developed from genes expressed in the leaves, which may have not been the primary target of selection in carrot. The genetic diversity of root-specific genes may therefore be reduced more dramatically in carrot domestication. Nevertheless, we can conclude that the genetic diversity of European cultivated carrot is significantly lower than that of wild carrot.

**Table 2:** **Genetic diversity estimates and Tajima’s D of cultivated carrot, wild carrot and wild carrot plus other wild** ***Daucus carota*** **subspecies**

	***H***_e_^1^	% polymorphic loci ^1^	***θ***_π_per kb ^1^	***θ***_w_per kb ^1^	Tajima’s D ^1^
Cultivated carrot	0.303 (0.288 - 0.317)	72.1 (69.2 - 74.7)	0.559 (0.532 - 0.584)	0.470 (0.452 - 0.487)	0.947 (0.846 - 1.042)
Wild carrot *Daucus carota* ssp. *carota*	0.349 (0.336 - 0.360)	84.0 (82.0 - 86.0)	0.643 (0.620 - 0.664)	0.548 (0.535 - 0.561)	0.869 (0.773 - 0.960)
Wild carrot plus other wild *D. carota* subspecies	0.344 (0.333 - 0.355)	84.3 (82.5 - 85.9)	0.635 (0.614 - 0.655)	0.550 (0.538 - 0.560)	0.776 (0.684 - 0.863)

^1^Values in parentheses indicate 95% confidence interval of estimates.

The domestication model we used is illustrated in Figure 2. For both the 622-SNP dataset without outgroup polarization and the 89-SNP dataset with outgroup polarization, the domestication model assuming asymmetric migration between cultivated and wild carrots is a much better fit to the data than models assuming symmetric migration or no migration (parameter estimates and likelihoods for both datasets and all three migration models are given in Additional file 3: Table S3). The maximum-likelihood estimates of parameters specified in Figure 2 with different datasets were virtually identical and here only the results based on the 622-SNP dataset are shown. Compared to the current effective population size of cultivated carrot *N*_*C*_, the bottleneck size was small: *N*_*B*_ = 0.0200*N*_*C*_ (95% CI: 0.0024 - 0.0346*N*_*C*_). However, the duration of the bottleneck *T*_*B*_ was also much shorter than the period of exponential growth *T* after the bottleneck: *T*_*B*_ = 0.0113*T* (95% CI: 0.0054 - 0.0195*T*), which may limit the loss of genetic diversity. Following the bottleneck, the effective population size of cultivated carrot increased exponentially to a present population size *N*_*C*_ of 0.1039*N*_*W*_ (95% CI: 0.0170 - 0.2508*N*_*W*_), which is smaller than the population size of wild carrot *N*_*W*_. The population growth took about *T* = 1.3138*N*_*W*_ (95% CI: 0.0964 - 2.0036*N*_*W*_) generations. During the population growth, asymmetric gene flow occurred between cultivated and wild carrots. The gene flow from cultivated to wild carrot *m*_*WC*_ was estimated at 0.1452/*N*_*W*_ (95% CI: 0.0002 - 0.3889/*N*_*W*_) while the gene flow from wild to cultivated carrot *m*_*CW*_ was 6.4537/*N*_*W*_ (95% CI: 2.0731 - 15.9550/*N*_*W*_). The significantly higher gene flow from wild to cultivated carrot may be the result of efforts to introduce genetic diversity from wild carrot germplasm into cultivated carrot for breeding purposes. Still, the final effective population size of cultivated carrot is significantly smaller than that of wild carrot and the genetic differentiation between them is high (*Fst* = 0.295). Moreover, as mentioned above, the Structure analyses provided some evidence of recent introgression, although cultivated and wild carrots remain in fairly distinct clusters (Figure 4). These results suggest that human selection had a strong impact on the genetic differentiation between cultivated and wild carrots.

Wild carrot is a widely distributed species native to temperate areas in the Mediterranean region, Europe and Western Asia [5]. Our results as well as those of Iorizzo et al. [10] suggest a single origin of cultivated carrot from wild carrot in Western and Central Asia, only a subset of the total genetic diversity in wild carrot. However, Iorizzo et al. [10] detected no reduction of genetic diversity in cultivated compared to wild carrots and proposed that the genetic bottleneck might be absent in carrot domestication. In our opinion, it is unlikely that the domestication of carrot did not go through a bottleneck at the beginning, and the results from our model simulations support this notion. Based on the simulations with different domestication models in our study, we propose another explanation of the relatively high genetic diversity maintaining in cultivated carrot. First, our model simulation suggests a small size of the domestication bottleneck but also a relatively short duration of the bottleneck, which implies a limited reduction in genetic diversity. Second, a relatively large amount of genetic diversity was recruited in cultivated carrot after the bottleneck through introgression from wild carrot. Because carrot is a predominantly outcrossing species, introgression may be relatively high between cultivated and wild carrots [12–14, 29, 30], either spontaneously or artificially, which is also supported by the results of model simulation above. For these reasons, the level of genetic diversity retained in cultivated carrot is higher than that found in other genome-wide studies of major crop species under strong pressure from bottlenecks and selection: for instance, both maize and rice, having about 57% (*θ*_*w*_ per kb) of the diversity in their progenitors [11, 31]. Our result is closer to that retained in the whole genome and in the protein coding sequences (CDS) of soybean, about 73.2% and 75.5% (*θ*_*w*_ per kb), respectively [25]. All major crops had much longer histories of domestication than carrot and the associated stronger effects of bottlenecks and selection may be responsible for the more severe loss of genetic diversity in the former.

### Putative genes under selection

**Figure 5:**
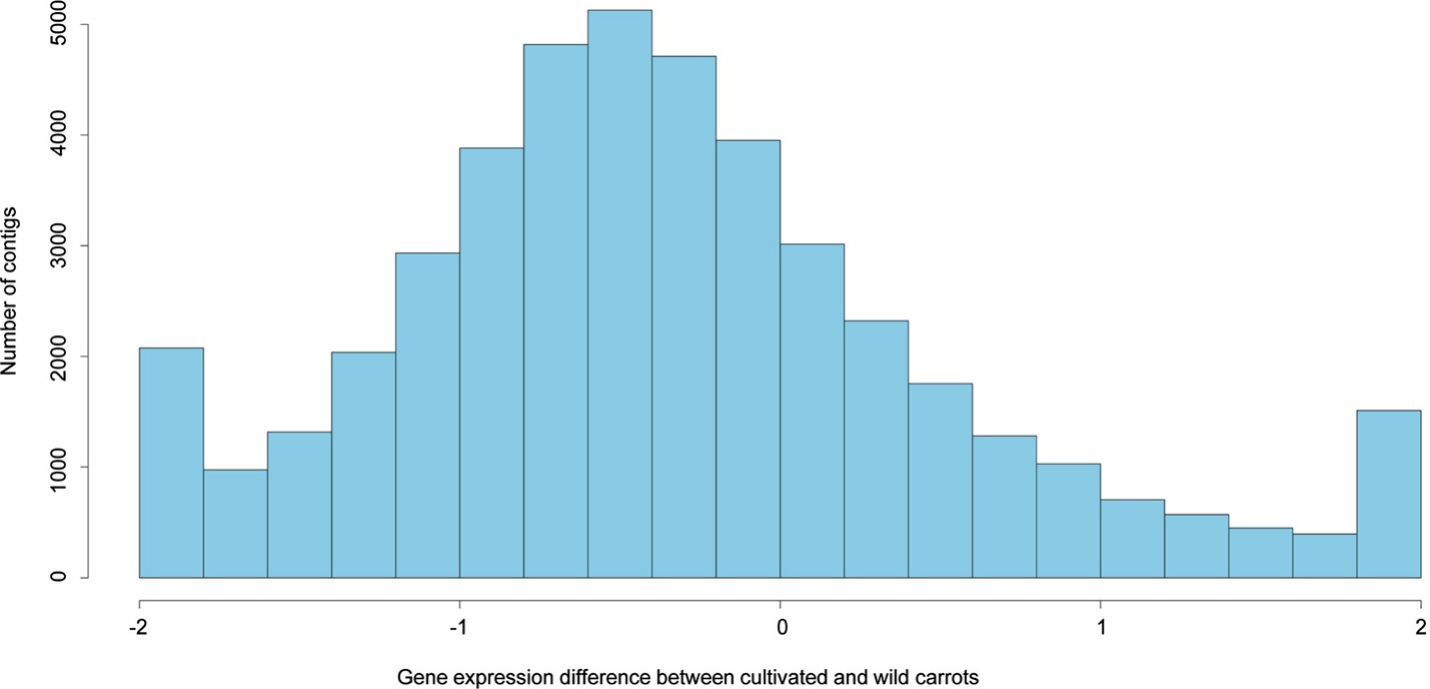
**Histogram of gene expression difference.** Gene expression difference between cultivated and wild carrots of a contig was calculated as (mean coverage of cultivated carrots - mean coverage of wild carrots) / (mean coverage of cultivated and wild carrots).

The histogram of gene expression difference between cultivated and wild carrots is shown in Figure 5. The contig number distribution in the histogram is shifted to the left, towards negative values of gene expression difference (Figure 5). The mean gene expression difference is -0.335 (95% CIs: -0.343 ~ -0.327), which is significantly lower than 0, showing more gene expressions down-regulated in cultivated carrot. Such results suggest that carrot domestication significantly altered gene expression patterns. The considerable increases in number of contigs at both ends of the histogram indicated that the expressions of some genes were radically different between cultivated and wild carrots (Figure 5). In particular, we found that the expressions of some genes were turned “on” or “off” in cultivated carrot compared to wild carrot. 174 contigs were expressed only in cultivated carrots (present in at least 5 of the 6 different cultivated carrot varieties studied) (Additional file 4), while 47 contigs were present only in the transcriptome of wild carrots (present in at least 4 of the 5 wild carrot populations) (Additional file 5). As indicated before, the mean coverage of all the contigs in the reference sequence is more or less the same for cultivated and wild carrots and the contigs in the reference generally have high coverage. Moreover, the data from each cultivated or wild carrot were the combination of 2–3 independent replicates. Therefore, the absence of reads from specifically all wild or all cultivated carrots at the same time is unlikely to be due to the variation in the read number of the various samples during sequencing. The histogram of gene expression difference between cultivated and wild carrots also strongly suggests that such radically different gene expression patterns were not due to chance (Figure 5). The observed unique expression pattern therefore indicated that the expression of these genes is radically different between cultivated and wild carrots. The special expression patterns of these genes may be related to key traits under strong selection during domestication and/or breeding processes (see below), which might be due to regulatory changes. Doebley et al. [1] expected that most domestication genes might be related to regulatory changes. Changes in regulatory genes while maintaining all other functional genes would lead to a smaller reduction in genetic diversity of the transcriptome than in studies based on whole genome sequencing data, because the latter includes also non-coding DNA that may be susceptible to genetic drift during the domestication bottlenecks.

Twenty-one of the unique expression contigs were found to have significant similarity to Genbank sequences (Table 3). Among these, important domestication gene candidates are the genes involved in water transport, the aquaporin genes. Cultivated carrot normally forms one large unbranched main root, while wild carrot has a long, thin and branched root with advanced lateral roots. A large amount of water is stored in cultivated carrot root. Such significant changes in domesticated carrot root might be associated with the changes in transcriptional regulation of aquaporin genes. Aquaporins are proteins that form water-selective channels, facilitating water flow across membranes [32]. A large proportion of aquaporin gene isoforms are predominantly expressed in roots and their activity can regulate the water flow across the root [32]. A tonoplast aquaporin gene was found to be generally expressed in cultivated carrots but not in wild carrots (Table 3), suggesting that transcriptional regulation of aquaporin genes was under selection during domestication.

**Table 3:** **Putative gene functions of unique expression contigs in either cultivated or wild carrots**

Putative functions	Contig ID	Length	Relative coverage ^1^		Significant alignments in NCBI nucleotide collection database ^2^				
			Cultivar	Wild	Accession	Score	E-value	Identities	Species
26S ribosomal RNA	190439	340	1.8 ± 0.5	0.0 ± 0.0	AY189100.1	111	2.E-21	97%	*Pimpinella saxifraga*
Alcohol dehydrogenase	146464	107	4.9 ± 0.8	0.0 ± 0.0	M86724.1	113	6.E-22	83%	*Lycopersicon esculentum*
Light harvesting protein	124533	392	33.4 ± 22.0	0.0 ± 0.0	Z75663.1	545	3.E-152	90%	*Apium graveolens*
	132345	187	19.4 ± 8.9	0.0 ± 0.0		221	2.E-54	86%	
	134075	113	182.1 ± 89.8	0.0 ± 0.0	DQ392956.1	154	2.E-34	90%	*Pachysandra terminalis*
	193833	365	38.9 ± 13.8	0.0 ± 0.0	GQ999612.1	398	1.E-107	84%	*Capsicum annuum*
Dihydroflavonol 4-reductase (DFR2)	82149	611	5.6 ± 1.7	0.0 ± 0.0	AF184272.1	441	1.E-120	83%	*Daucus carota*
	168644	116	17.5 ± 3.3	0.0 ± 0.0		174	2.E-40	93%	
Glycine-rich protein	134512	168	2.9 ± 1.1	0.0 ± 0.0	X58146.1	104	3.E-19	98%	*Daucus carota*
Peptidyl-prolyl cis-trans isomerase B	187919	574	25.8 ± 8.0	0.0 ± 0.0	XM_002511947.1	255	8.E-65	81%	*Ricinus communis*
Phosphatidic acid phosphatase alpha	117946	571	9.8 ± 3.6	0.0 ± 0.0	EF076031.1	165	1.E-37	80%	*Vigna unguiculata*
Phosphoribulokinase	116742	1538	16.6 ± 10.2	0.0 ± 0.0	XM_002326536.1	1207	0	81%	*Populus trichocarpa*
Photosystem I reaction center subunit	193998	320	21.4 ± 4.0	0.0 ± 0.0	XM_002521115.1	214	3.E-52	83%	*Ricinus communis*
	194107	269	15.5 ± 7.9	0.0 ± 0.0	M83119.1	284	2.E-73	83%	*Flaveria trinervia*
Plastid division regulator MinD mRNA	208192	122	16.7 ± 5.3	0.0 ± 0.0	DQ118107.1	143	4.E-31	86%	*Populus tomentosa*
Ribosomal protein S3	170401	142	3.2 ± 1.2	0.0 ± 0.0	GU351776.1	122	1.E-24	96%	*Pittosporum tobira*
Tonoplast aquaporin 1;1	146558	118	23.5 ± 7.0	0.0 ± 0.0	FJ861240.1	111	2.E-21	95%	*Daucus carota*
*Daucus carota* major allergen isoform Dau c1.0201	186900	102	0.0 ± 0.0	52.1 ± 33.9	AF456481.1	136	3.E-29	98%	*Daucus carota*
	207957	201	0.0 ± 0.0	209.8 ± 58.0		96.9	3.E-17	98%	
Phloem protein 2-2	159264	157	0.0 ± 0.0	28.1 ± 17.8	AY114140.1	113	4.E-22	96%	*Apium graveolens* var*. dulce*
Receptor protein kinase	232664	128	0.0 ± 0.0	32.0 ± 9.2	XM_002509756.1	127	2.E-26	82%	*Ricinus communis*

^1^Relative coverage = Mean coverage of a contig/Mean coverage of all contigs × 100% (Mean ± Standard Error %).

^2^Only the accessions with a score ≥96.9, E-value ≤3E-17, and Identities ≥80% are shown.

An interesting finding is the activated expression of the light-harvesting complex protein of photosystem II (LHC-II) genes (*Lhcb*-like) in cultivated carrot roots (Table 3). LHC-II proteins are chloroplast membrane proteins encoded by a nuclear multigene family. They bind mainly chlorophyll, and therefore are often referred to as chlorophyll a/b binding proteins [33–35]. They play important roles in photosynthesis, especially in the regulation of energy flow between photosystem I and II and control of the dissipation of excess energy under light stress [34, 35]. LHC-II proteins also bind yellow or orange carotenoids, in particular lutein, zeaxanthin, violaxanthin, neoxanthin and β-carotene [34, 35]. The expression of *Lhcb* genes appears to be regulated by light, and plants grown in darkness contain a very low amount of *Lhcb* mRNA [33, 34]. Carotenoid-deficient leaves contain only trace amounts of *Lhcb* mRNA, suggesting that carotenoid biosynthesis and *Lhcb* gene expression are directly related [33]. The *Lhcb* genes were thought to be silenced in roots. The high expression of *Lhcb* genes that we have found in cultivated carrot roots but not in wild carrot roots may be related to the high carotenoid accumulation in the former. Cultivated carrot is renowned for the high carotenoid content of its roots (xanthophylls for yellow, α- and β-carotene for orange roots), while wild carrot contains only traces of carotenoids (mainly xanthophylls) in roots [5]. The activated expression of *Lhcb* genes may lead to the production of LHC-II proteins, and the binding to carotenoids of LHC-II may stimulate the accumulation of carotenoids in cultivated carrot. Carotenoid biosynthesis and the binding of carotenoids to LHC-II occur within plastids. Thus, the expression of *Lhcb* genes may be related to the differentiation of plastid to chromoplast in cultivated carrot roots [33, 36]. A plastid division regulator MinD gene was also found to be activated only in cultivated carrot roots (Table 3). The expression of the MinD gene may help to increase the amount of chromoplast, promote the expression of *Lhcb* genes and encourage the accumulation of carotenoids as shown by Galpaz et al. (2008) in tomato [37]. Further studies are required to figure out the roles these genes played in the accumulation of carotenoids in carrot roots.

Putative allergen-related protein genes were expressed only in wild carrot roots (Table 3). The allergen-related proteins are presumed to be involved in plant defenses against microbial pathogens and abiotic stresses, but may also cause allergenic reactions in humans [38]. The silencing of such genes in cultivated carrot may be the results of human selection for reducing allergy in cultivated carrot and/or due to different responses to stresses.

## Conclusions

We studied carrot domestication based on transcriptome analyses of a diverse set of cultivated carrot, wild carrot and other wild *D. carota* subspecies. The results support the hypothesis that eastern-type carrot may have been domesticated from wild carrots in Western Asia. In addition to wild carrot, other wild *D. carota* subspecies may have contributed to the origin of cultivated carrots. Western-type orange carrot may originate from eastern carrot though introgression from wild carrots may also have played a role in the process. The genetic bottleneck during domestication reduced the genetic diversity in cultivated carrot, but a large amount of genetic diversity is still present in cultivated carrot. Model simulations support an important role of introgression from wild carrot in the increase of genetic diversity of cultivated carrot after the bottleneck, by breeding and/or through frequent gene flow between cultivated and wild carrots. Still, the high genetic differentiation between cultivated and wild carrots indicates the strong effects of selection. Our study demonstrated that high-throughput transcriptome sequencing of diverse cultivars and wild accessions may be very helpful in identifying functional genes under selection. Results of gene expression analysis suggest that carrot domestication significantly altered gene expression patterns by generally down-regulating the gene expressions in cultivated carrot roots. In addition, the expressions of some genes were radically different between cultivated and wild carrots. We found 174 contigs that were expressed only in cultivated carrot roots and 47 only in wild carrot roots. Transcriptional changes may be predominant among the major putative domestication genes controlling the differences between cultivated and wild carrots. Many of these genes are still unknown, however, and these require further analysis. In future studies, special attention shall be devoted to functional analysis of the genes under selection identified in the present study and to discovering the detailed molecular mechanisms of those genes in changing root traits in carrot.

## Availability of supporting data

The data sets supporting the results of this article are included within the article (and its additional files), RNA-seq data are available in the ArrayExpress Archive database of functional genomics experiments at the European Bioinformatics Institute (EBI) under accession E-MTAB-1340 (http://www.ebi.ac.uk/arrayexpress/experiments/E-MTAB-1340/), the phylogenetic tree and associated data matrix are available in TreeBASE (Accession URL: http://purl.org/phylo/treebase/phylows/study/TB2:S16441?format=html).

## Electronic supplementary material

Additional file 1: Table S1: Additional set of cultivated carrots, wild carrots, other wild *Daucus carota* subspecies and wild *Daucus* species used in the study. (DOC 168 KB)

Additional file 2: Table S2: Primers for validated SNPs in carrot transcriptome. (XLS 178 KB)

Additional file 3: Table S3: Parameter values, 95% confidence intervals, and likelihoods for both datasets and the three examined models of migration between cultivated and wild carrots. For both datasets the asymmetric migration model has a significantly higher likelihood than either the symmetric migration model or the no migration model (*P* <0.0001). (DOC 41 KB)

Additional file 4: **Unique expression contigs in cultivated carrot.** (ZIP 22 KB)

Additional file 5: **Unique expression contigs in wild carrot.** (ZIP 4 KB)
