# Supplementary material for: New insights into domestication of carrot from root transcriptome analyses
Source: BMC Genomics. 2014 Oct 14;15(1):895. doi: 10.1186/1471-2164-15-895 (PMC4213543; doi:10.1186/1471-2164-15-895)
Supplement: Supplementary file 1 — Additional file 1: Table S1: Additional set of cultivated carrots, wild carrots, other wild Daucus carota subspecies and wild Daucus species used in the study. (DOC 168 KB) [file 12864_2014_6606_MOESM1_ESM.doc]

**Table S1 Additional set of cultivated carrots, wild carrots, other wild *Daucus carota* subspecies and wild *Daucus* species used in the study.**

| **Label^1^** | **Accession number^2^** | **Accession name** | **Species name** | **Root type^3^** | **Country^4^** |
| --- | --- | --- | --- | --- | --- |
| CHR01 | 3834 | Flakkee French Strain | *Daucus carota* L. ssp. *sativus* (Hoffm.) Arc. | Flakkee | France |
| CHR02 | 3898 | Cluseed New Model | *D. carota* L. ssp. *sativus* (Hoffm.) Arc. | Chantenay | UK |
| CHR03 | 3906 | Oxheart | *D. carota* L. ssp. *sativus* (Hoffm.) Arc. | Oxheart | USA |
| CHR04 | 3921 | Gelbe Rheinische | *D. carota* L. ssp. *sativus* (Hoffm.) Arc. | Yellow Belgian | Germany |
| CHR05 | 3929 | St. Valery | *D. carota* L. ssp. *sativus* (Hoffm.) Arc. | Long Orange | Netherlands |
| CHR06 | 3957 | Nantes Marko | *D. carota* L. ssp. *sativus* (Hoffm.) Arc. | Nantes | Netherlands |
| CHR07 | 3971 | Champion Scarlet Horn | *D. carota* L. ssp. *sativus* (Hoffm.) Arc. | Early Short Horn | UK |
| CHR08 | 3990 | Blanche A Collet Vert Hors Terre | *D. carota* L. ssp. *sativus* (Hoffm.) Arc. | Yellow Belgian | France |
| CHR09 | 3991 | Carentan | *D. carota* L. ssp. *sativus* (Hoffm.) Arc. | Nantes | France |
| CHR10 | 4005 | Kuroda Gosun | *D. carota* L. ssp. *sativus* (Hoffm.) Arc. | Kuroda | Japan |
| CHR11 | 5128 | Danvers Red Cored | *D. carota* L. ssp. *sativus* (Hoffm.) Arc. | Danvers | USA |
| CHR12 | 5477 | Amsterdam Forcing | *D. carota* L. ssp. *sativus* (Hoffm.) Arc. | Amsterdam Forcing | UK |
| CHR13 | 5595 | Danvers Danro Rs | *D. carota* L. ssp. *sativus* (Hoffm.) Arc. | Danvers | Netherlands |
| CHR14 | 5596 | Paris Market | *D. carota* L. ssp. *sativus* (Hoffm.) Arc. | Paris Market | Netherlands |
| CHR15 | 5649 | Topweight | *D. carota* L. ssp. *sativus* (Hoffm.) Arc. | Flakkee | Australia |
| CHR16 | 5816 | Long Imperator 58 | *D. carota* L. ssp. *sativus* (Hoffm.) Arc. | Imperator | USA |
| CHR17 | 6089 | Early Nantes | *D. carota* L. ssp. *sativus* (Hoffm.) Arc. | Nantes | UK |
| CHR18 | 6091 | De La Halle | *D. carota* L. ssp. *sativus* (Hoffm.) Arc. | Nantes | France |
| CHR19 | 6100 | James Scarlet Intermediate | *D. carota* L. ssp. *sativus* (Hoffm.) Arc. | Late Half Long Horn | UK |
| CHR20 | 6102 | Long Red Surrey | *D. carota* L. ssp. *sativus* (Hoffm.) Arc. | Long Orange | UK |
| CHR21 | 6162 | Early French Frame | *D. carota* L. ssp. *sativus* (Hoffm.) Arc. | Paris Market | UK |
| CHR22 | 6490 | Early Scarlet Horn | *D. carota* L. ssp. *sativus* (Hoffm.) Arc. | Early Short Horn | UK |
| CHR23 | 6491 | Guerande | *D. carota* L. ssp. *sativus* (Hoffm.) Arc. | Oxheart | UK |
| CHR24 | 6767 | Duwicker | *D. carota* L. ssp. *sativus* (Hoffm.) Arc. | Early Short Horn | Germany |
| CHR25 | 7134 | Cold King | *D. carota* L. ssp. *sativus* (Hoffm.) Arc. | Chantenay | France |
| CHR26 | 8720 | White Belgian | *D. carota* L. ssp. *sativus* (Hoffm.) Arc. | Yellow Belgian | UK |
| CHR27 | 10110 | Danvers Half Long Danvers 126 | *D. carota* L. ssp. *sativus* (Hoffm.) Arc. | Danvers | UK |
| CHR28 | 10127 | Berlicum Belinda | *D. carota* L. ssp. *sativus* (Hoffm.) Arc. | Berlicum | UK |
| CHR29 | 11145 | Imperator | *D. carota* L. ssp. *sativus* (Hoffm.) Arc. | Imperator | Netherlands |
| CHR30 | 11146 | Gelbe Wortel | *D. carota* L. ssp. *sativus* (Hoffm.) Arc. | Yellow Belgian | Netherlands |
| CHR31 | 11147 | Amsterdammer Bak Long | *D. carota* L. ssp. *sativus* (Hoffm.) Arc. | Amsterdam Forcing | Netherlands |
| CHR32 | 11150 | Chantenay Kort | *D. carota* L. ssp. *sativus* (Hoffm.) Arc. | Chantenay | Netherlands |
| CHR33 | 11151 | Flakkee Grof | *D. carota* L. ssp. *sativus* (Hoffm.) Arc. | Flakkee | Netherlands |
| CHR34 | 11157 | Berlicum Normaal | *D. carota* L. ssp. *sativus* (Hoffm.) Arc. | Berlicum | Netherlands |
| CHR35 | 12400 | Altringham | *D. carota* L. ssp. *sativus* (Hoffm.) Arc. | Altringham | UK |
| CHR36 | 12480 | Altringham Large Red | *D. carota* L. ssp. *sativus* (Hoffm.) Arc. | Altringham | Czech Republic |
| CHR37 | 12845 | Kuroda 5 Sun | *D. carota* L. ssp. *sativus* (Hoffm.) Arc. | Kuroda | Japan |
| **CHR38** | 6755 | Pusa Kesar | *D. carota* L. ssp. *sativus* (Hoffm.) Arc. | − | India |
| **CHR39** | 6760 | Pink Selection | *D. carota* L. ssp. *sativus* (Hoffm.) Arc. | − | China |
| **CHR40** | 7126 | Tropical | *D. carota* L. ssp. *sativus* (Hoffm.) Arc. | Chantenay | Brazil |
| **CHR41** | 10111 | Early Nantes | *D. carota* L. ssp. *sativus* (Hoffm.) Arc. | Nantes | India |
| **CHR42** | 10246 | Long Red | *D. carota* L. ssp. *sativus* (Hoffm.) Arc. | − | Ethiopia |
| **CHR43** | 10266 | Zardak | *D. carota* L. ssp. *sativus* (Hoffm.) Arc. | − | Afghanistan |
| **CHR44** | 10344 | Gajer | *D. carota* L. ssp. *sativus* (Hoffm.) Arc. | − | Pakistan |
| **CHR45** | 10506 | Sian Chi-Tou | *D. carota* L. ssp. *sativus* (Hoffm.) Arc. | − | China |
| **CHR46** | 10508 | Red Carrot | *D. carota* L. ssp. *sativus* (Hoffm.) Arc. | − | China |
| **CHR47** | 11201 | Afghan Purple | *D. carota* L. ssp. *sativus* (Hoffm.) Arc. | − | USA |
| **CHR48** | 10264 | Garga Serk | *D. carota* L. ssp. *sativus* (Hoffm.) Arc. | − | Pakistan |
| **CBW** | − | White Berlicum | *D. carota* L. ssp. *sativus* (Hoffm.) Arc. | Berlicum | Netherlands |
| WHR01 | 6666 | Wild carrot | *D. carota* L. ssp. *carota* (Hoffm.) Arc. | Wild | Ireland |
| WHR02 | 6667 | Wild *D. carota* subspecies | *D. carota* L. ssp. *azoricus* Franco | Wild | Spain |
| WHR03 | 6668 | Wild *D. carota* subspecies | *D. carota* L. ssp. *drepanensis* (Arc.) Heywood | Wild | UK |
| WHR04 | 6673 | Wild carrot | *D. carota* L. ssp. *carota* (Hoffm.) Arc. | Wild | UK |
| WHR05 | 7160 | Wild *D. carota* subspecies | *D. carota* L. ssp. *gadecaei* (Rony & Camus) Heywood | Wild | France |
| WHR06 | 7388 | Wild *D. carota* subspecies | *D. carota* L. ssp. *maritimus* (Lam.) Batt. | Wild | France |
| WHR07 | 8252 | Wild *D. carota* subspecies | *D. carota* L. ssp. *hispanicus* (Gouan) Thell. | Wild | Germany |
| WHR08 | 8715 | Wild *D. carota* subspecies | *D. carota* L. ssp. *gummifer* Hooker fil. | Wild | UK |
| WHR09 | 9202 | Wild *D. carota* subspecies | *D. carota* L. ssp. *gadecaei* (Rony & Camus) Heywood | Wild | France |
| WHR10 | 9216 | Wild *D. carota* subspecies | *D. carota* L. ssp. *maritimus* (Lam.) Batt. | Wild | Portugal |
| WHR11 | 9217 | Wild *D. carota* subspecies | *D. carota* L. ssp. *maritimus* (Lam.) Batt. | Wild | Spain |
| WHR12 | 9289 | Wild *D. carota* subspecies | *D. carota* L. ssp. *gummifer* Hooker fil. | Wild | Germany |
| WHR13 | 9290 | Wild *D. carota* subspecies | *D. carota* L. ssp. *maritimus* (Lam.) Batt. | Wild | Germany |
| WDA01 | DAU 126 | Wild carrot | *D. carota* L. ssp. *carota* (Hoffm.) Arc. | Wild | Germany |
| WDA02 | DAU 214 | Wild carrot | *D. carota* L. ssp. *carota* (Hoffm.) Arc. | Wild | Uzbekistan |
| WDA03 | DAU 215 | Wild carrot | *D. carota* L. ssp. *carota* (Hoffm.) Arc. | Wild | Italy |
| WDA04 | DAU 216 | Wild carrot | *D. carota* L. ssp. *carota* (Hoffm.) Arc. | Wild | Austria |
| WDA05 | DAU 258 | Wild carrot | *D. carota* L. ssp. *carota* (Hoffm.) Arc. | Wild | Italy |
| WDA06 | DAU 261 | Wild carrot | *D. carota* L. ssp. *carota* (Hoffm.) Arc. | Wild | Iraq |
| WDA07 | DAU 339 | Wild carrot | *D. carota* L. ssp. *carota* (Hoffm.) Arc. | Wild | Azerbaijan |
| WDA08 | DAU 354 | Wild carrot | *D. carota* L. ssp. *carota* (Hoffm.) Arc. | Wild | Azerbaijan |
| WDA09 | DAU 384 | Wild carrot | *D. carota* L. ssp. *carota* (Hoffm.) Arc. | Wild | Iran |
| WDA10 | DAU 397 | Wild carrot | *D. carota* L. ssp. *carota* (Hoffm.) Arc. | Wild | Libya |
| WDA11 | DAU 418 | Wild carrot | *D. carota* L. ssp. *carota* (Hoffm.) Arc. | Wild | Belgium |
| WDA12 | DAU 433 | Wild carrot | *D. carota* L. ssp. *carota* (Hoffm.) Arc. | Wild | Portugal |
| WDA13 | DAU 497 | Wild carrot | *D. carota* L. ssp. *carota* (Hoffm.) Arc. | Wild | Austria |
| WDA14 | DAU 127 | Wild *D. carota* subspecies | *D. carota* L. ssp. *commutatus* (Paol.) Thell. | Wild | Italy |
| WDA15 | DAU 236 | Wild *D. carota* subspecies | *D. carota* L. ssp. *commutatus* (Paol.) Thell. | Wild | France |
| WDA16 | DAU 486 | Wild *D. carota* subspecies | *D. carota* L. ssp. *commutatus* (Paol.) Thell. | Wild | Italy |
| WDA17 | DAU 496 | Wild *D. carota* subspecies | *D. carota* L. ssp. *gadecaei* (Rony & Camus) Heywood | Wild | France |
| WDA18 | DAU 134 | Wild *D. carota* subspecies | *D. carota* L. ssp. *gummifer* Hooker fil. | Wild | France |
| WDA19 | DAU 241 | Wild *D. carota* subspecies | *D. carota* L. ssp. *gummifer* Hooker fil. | Wild | France |
| WDA20 | DAU 245 | Wild *D. carota* subspecies | *D. carota* L. ssp. *hispanicus* (Gouan) Thell. | Wild | Spain |
| WDA21 | DAU 243 | Wild *D. carota* subspecies | *D. carota* L. ssp. *hispidus* (Arc.) Heywood | Wild | Greece |
| WDA22 | DAU 508 | Wild *D. carota* subspecies | *D. carota* L. ssp. *hispidus* (Arc.) Heywood | Wild | Italy |
| WDA23 | DAU 217 | Wild *D. carota* subspecies | *D. carota* L. ssp. *major* (Vis.) Arc. | Wild | France |
| WDA24 | DAU 240 | Wild *D. carota* subspecies | *D. carota* L. ssp. *major* (Vis.) Arc. | Wild | France |
| WDA25 | DAU 244 | Wild *D. carota* subspecies | *D. carota* L. ssp. *maritimus* (Lam.) Batt. | Wild | UK |
| WDA26 | DAU 218 | Wild *D. carota* subspecies | *D. carota* L. ssp. *maximus* (Desf.) Ball. | Wild | Greece |
| WDA27 | DAU 237 | Wild *D. carota* subspecies | *D. carota* L. ssp. *maximus* (Desf.) Ball. | Wild | Croatia |
| WDA28 | DAU 239 | Wild *D. carota* subspecies | *D. carota* L. ssp. *maximus* (Desf.) Ball. | Wild | Greece |
| WDA29 | DAU 246 | Wild *D. carota* subspecies | *D. carota* L. ssp. *maximus* (Desf.) Ball. | Wild | Italy |
| WDA30 | DAU 249 | Wild *D. carota* subspecies | *D. carota* L. ssp. *maximus* (Desf.) Ball. | Wild | Tunisia |
| WDA31 | DAU 250 | Wild *D. carota* subspecies | *D. carota* L. ssp. *maximus* (Desf.) Ball. | Wild | Spain |
| WDA32 | DAU 252 | Wild *D. carota* subspecies | *D. carota* L. ssp. *maximus* (Desf.) Ball. | Wild | Spain |
| WDA33 | DAU 257 | Wild *D. carota* subspecies | *D. carota* L. ssp. *maximus* (Desf.) Ball. | Wild | Slovenia |
| WDA34 | DAU 503 | Wild *D. carota* subspecies | *D. carota* L. ssp. *maximus* (Desf.) Ball. | Wild | Cyprus |
| **WDA35** | DAU 123 | Wild *Daucus* | *D. aureus* Desf. | Wild | Israel |
| **WDA36** | DAU 274 | Wild *Daucus* | *D. muricatus* L. | Wild | Morocco |
| **WDA37** | DAU 504 | Wild *Daucus* | *D. muricatus* L. | Wild | Portugal |
| **WDA38** | DAU 484 | Wild *Daucus* | *D. broteri* Ten. | Wild | Turkey |
| **WDA39** | DAU 531 | Wild *Daucus* | *D. broteri* Ten. | Wild | Cyprus |
| **WDA40** | DAU 523 | Wild *Daucus* | *D. guttatus* Sibth. & Sm. | Wild | Greece |
| **WAL1** | − | Wild carrot | *D. carota* L. ssp. *carota* (Hoffm.) Arc. | Wild | Netherlands |
| **WA1** | − | Wild carrot | *D. carota* L. ssp. *carota* (Hoffm.) Arc. | Wild | Netherlands |
| **WB1** | − | Wild carrot | *D. carota* L. ssp. *carota* (Hoffm.) Arc. | Wild | Netherlands |

**^1^**Those indicated with bold face (21 accessions) were genotyped with 89 SNPs, a subset of the 622 SNPs used to genotype all other carrot samples in the table.

**^2^**Accession numbers beginning with “DAU” indicate plant materials from The Genebank of the Leibniz Institute of Plant Genetics and Crop Plant Research (IPK Gatersleben) in Germany. Others represent plant materials from Warwick Genetic Resources Unit in the United Kingdom. Those indicated with “−” were sampled by the authors and only DNA samples are available.

**^3^**The root types of those indicated with “−” are unknown.

^4^Name of the country where the samples were originally collected or derived.
