# Supplementary material for: New insights into domestication of carrot from root transcriptome analyses
Source: BMC Genomics. 2014 Oct 14;15(1):895. doi: 10.1186/1471-2164-15-895 (PMC4213543; doi:10.1186/1471-2164-15-895)
Supplement: Supplementary file 3 — Additional file 3: Table S3: Parameter values, 95% confidence intervals, and likelihoods for both datasets and the three examined models of migration between cultivated and wild carrots. For both datasets the asymmetric migration model has a significantly higher likelihood than either the symmetric migration model or the no migration model (P <0.0001). (DOC 41 KB) [file 12864_2014_6606_MOESM3_ESM.doc]

**Table S3 Parameter values, 95% confidence intervals, and likelihoods for both datasets and the three examined models of migration between cultivated and wild carrots. For both datasets the asymmetric migration model has a significantly higher likelihood than either the symmetric migration model or the no migration model (*P* < 0.0001).**

| **Dataset** | **Migration model** | ***N_B_*^1^** | ***T_B_*^2^** | ***N_C_*^3^** | ***T*^4^** | ***m_WC_*^5^** | ***m_CW_*^6^** | **Ln(L)^7^** |
| --- | --- | --- | --- | --- | --- | --- | --- | --- |
| 622 SNPs without outgroup polarization | Asymmetric | 0.0200*N_C_* (0.0024-0.0346*N_C_*) | 0.0113*T* (0.0054-0.0195*T*) | 0.1039*N_W_* (0.0170-0.2508*N_W_*) | 1.3138*N_W_* (0.0964-2.0036*N_W_*) | 0.1452/*N_W_* (0.0002-0.3889/*N_W_*) | 6.4537/*N_W_* (2.0731-15.9550/*N_W_*) | -373.771 |
|  | Symmetric | 0.0931*N_C_* (0.0106-0.4805*N_C_*) | 0.0103*T* (0.0050-0.0173*T*) | 0.5565*N_W_* (0.1070-0.9997*N_W_*) | 1.4722*N_W_* (0.1111-2.0008*N_W_*) | 3.8055/*N_W_*  (0.3337-18.3134/*N_W_*) | | -438.520 |
|  | No migration | 0.2262*N_C_* (0.0029-0.9847*N_C_*) | 0.0077*T* (0.0026-0.0188*T*) | 0.3130*N_W_* (0.0090-0.9997*N_W_*) | 0.1918*N_W_* (0.0022-1.1834*N_W_*) | 0 | | -557.084 |
| 89 SNPs with outgroup polarization | Asymmetric | 0.0177*N_C_* (0.0060-0.0334*N_C_*) | 0.0107*T* (0.0051-0.0197*T*) | 0.1142*N_W_* (0.0411-0.2154*N_W_*) | 1.4700*N_W_* (0.5089-1.997*N_W_*) | 0.1077/*N_W_* (0.0027-0.2551/*N_W_*) | 4.0043/*N_W_* (2.0198-8.5191/*N_W_*) | -172.260 |
|  | Symmetric | 0.0495*N_C_* (0.0092-0.2971*N_C_*) | 0.0107*T* (0.0052-0.0191*T*) | 0.4923*N_W_* (0.1735-0.9995*N_W_*) | 1.6358*N_W_* (0.1937-2.0028*N_W_*) | 4.8002/*N_W_* (0.3087-24.9007/*N_W_*) | | -190.576 |
|  | No migration | 0.1724*N_C_* (0.0022-0.9509*N_C_*) | 0.0090*T* (0.0018-0.0225*T*) | 0.4292*N_W_* (0.0340-1.0014*N_W_*) | 0.2694*N_W_* (0.0023-1.5043*N_W_*) | 0 | | -220.116 |

^1^*N_B_* is the size of domestication bottleneck. ^2^*T_B_* is the duration (generation) of the bottleneck. ^3^*N_C_* is the present population size of cultivated carrot. ^4^*T* is the number of generations after the bottleneck till present. ^5^*m_WC_* is the migration rate from cultivated to wild carrot. ^6^*m_CW_* is the migration rate from wild to cultivated carrot. ^7^Log-likelihood of the migration model.
